# Editorial: Psychosocial interventions in psychotic illnesses

**DOI:** 10.3389/fpsyt.2022.1092976

**Published:** 2022-12-14

**Authors:** Padmavati Ramachandran, Swapna Kamal Verma, Swaran Preet Singh

**Affiliations:** ^1^Schizophrenia Research Foundation, Chennai, India; ^2^Institute of Mental Health and Duke NUS Medical School, Singapore, Singapore; ^3^Division of Health Sciences, Warwick Medical School, Faculty of Science, Engineering and Medicine, University of Warwick, Coventry, United Kingdom

**Keywords:** psychosocial interventions, replication, innovation, early psychoses, chronic psychoses

Evidence indicates that pharmacotherapy is the mainstay of treatment, both in early and chronic psychosis. Despite clinical improvement, many people with psychosis develop disabilities related to domains of negative, physical health and mood symptoms, cognitive dysfunction, and socioeconomic disadvantages, through all stages of psychoses. This implies a key need for psychosocial interventions. Initiation of combined drug and psychosocial treatments plays a significant part in the early phase of psychosis (1). Several meta-analyses have highlighted the need to address the complex health, social, and economic needs of those diagnosed with a chronic and highly disabling illness such as schizophrenia (2). A recent systematic review of recovery-oriented interventions delivered in pro-recovery and non-stigmatizing contexts has been reported to share several critical mechanisms, that propel service users toward recovery (3).

This collection of articles aimed to bring together various psychosocial interventions across the course and chronicity of psychotic illnesses. The objective was to understand the innovations and adaptations of interventions both in the early phase as well as the chronic phase of the illness. It was also felt necessary to appreciate the application of these multi-module interventions in real-world settings. This volume brings together various interventions from different parts of the globe.

## Interventions that are well-established and have shown effectiveness based on previous evidence

### Getting persons with psychoses back to work

There is a noteworthy relationship between symptoms, cognition, social functions, and employment (4–7). Several social determinants such as income, daily life, housing, or social support for patients with psychosis can be helped through employment (6). Patients in employment are also reported to manage their illnesses better (6). In LMIC, it becomes even more relevant to help persons with psychoses find and maintain meaningful employment. “Individual placement and Support (IPS)” program is an evidence-based intervention that helps persons with psychoses to succeed in competitive employment, in integrated work settings, supervised and paid by the business (8). Literature shows that people with mental disorders who gain employment experience broad benefits, including enhanced self-esteem, community integration, social support, and quality of life (9). Although substantial evidence supports the finding that IPS is superior to conventional methods in improving employment rates, keeping patients in competitive jobs over longer periods and with higher salaries (10, 11), may not persist beyond the intervention period.

Pichler et al. investigating the long-term employment status of individuals with mental illnesses, who have received IPS, make important observations. In the randomized controlled ZhEPP trial, 250 Swiss disability pensioners with mental illnesses were randomly allocated into either IPS intervention or treatment as usual group (TAU). The trial demonstrated that IPS was better than standard approaches for pensioners with mental illnesses to get jobs in the competitive employment space. Six years after the start of the trial, Pichler et al., interviewed 114 individuals on their employment status, job tenure, workload, and salaries. The IPS group had higher employment rates (40%) when compared to TAU (28%), at 24 months. However, no differences between the groups were noted at 72-month follow-up assessments. These findings imply the need for longitudinal studies to examine the adaptation and sustainability of interventions like IPS.

### The role of physical activity and yoga

Less than half of people with mental illnesses engage in the recommended 150 min of moderate-vigorous physical activity per week and most have low levels of cardiorespiratory fitness (12). The high incidence of cardiovascular diseases and premature mortality in patients with schizophrenia has been attributed to a sedentary lifestyle, low physical activity, obesity, and metabolic syndrome (13). In 2019, the Lancet Psychiatry Commission for protecting the physical health of people with mental illness, recommended collaborative care for physical health problems in persons with serious mental disorders (14). Is it feasible to incorporate physical health care within community care mental health services? Fibbins et al. report on the feasibility of integrating an exercise clinic, within a mental health service for people with serious mental disorders in Sydney, Australia. Using a single-site, open-trial methodology, an accredited exercise physiologist delivered individually tailored physical activity interventions in a part-time exercise physiology clinic. Outcome measures included body mass index, cardiovascular indicators, and a self-report physical activity. An average of 1 weekly exercise session was delivered to a total of 84 mental health service users. The study demonstrated a statistically significant increase in physical activity and a decrease in inactivity. The researchers concluded that exercise clinics were feasible within mental health services. They recommended that such a service if incorporated within a routine care service, would help address barriers like staff culture, finance, and resources to integrating physical activity as part of standard care.

Yoga, as an adjunct intervention, has been shown to significantly improve the severity of psychopathology and functioning in patients with schizophrenia (15, 16). Specific yoga modules for schizophrenia have been developed and validated (17) and have demonstrated a significant reduction in negative symptoms and emotion recognition deficits as well as improvement in real-life functioning (18–21). An expert panel of the National Institute of Health and Care Excellence (NICE) (UK) in the 2014 guidelines for Schizophrenia included evidence for use of yoga in schizophrenia, as “high quality” and recommended the intervention as a complementary treatment in schizophrenia.

However, few studies have qualitatively examined the patient perspectives on the experiences of Yoga. Schulze et al. have explored the experiences of participants in the Yoga-based Group Intervention (YoGI) for in-patients with schizophrenia spectrum disorders (SSD) in Germany using qualitative methods. Interviews with in-patients explored the personal experiences of taking part in YoGI. The transcribed interview data were coded and analyzed using an inductive thematic approach. The respondents experienced interconnectedness, were mindful of their limitations, and were able to adapt to the practice. The persistent practice of the asanas resulted in an experience of confidence and relaxation. Increased awareness of self resulted in the participants becoming aware of imminent as well as reduced severity of symptoms. The researchers opined that YoGI showed several promising benefits for in-patients with SSD and recommended that future studies should focus on sustainability and transfer of methods to routine use.

### A dedicated safe space

Clubhouses are programs that help people with mental illness establish a daily routine, social and affective connectedness, in a context other than the usual medical care settings. People are treated as individuals, than “patients” (22). Significant positive psychosocial outcomes and enduring resiliencies for persons with severe mental illness have been demonstrated in such programs. Clubhouse models provide a safe environment, caring relationships, and supported employment activities. The lack of an integrated psychiatry clinic and the risk of dependence on the services are some criticisms. Despite this, it has been well acknowledged that the Clubhouse model is an important influence in the field of psychiatric rehabilitation (22). However, the complexity of the program is a challenge to study the outcomes and impact.

Yan et al. undertook a systematic review and a meta-analysis of the clubhouse model of psychiatric rehabilitation in China. They investigated the role of the model in promoting symptom remission and functional recovery of persons with schizophrenia. Data extracted from 7 databases were subjected to quality assessment, data synthesis, and subgroup analysis. Both randomized and non-randomized studies were included. The overall results showed that psychopathology, social and occupational functioning and quality of life improved significantly and a reduction in family burden was noted. The researchers recommended that the clubhouse model may be useful to meet the urgent need for improving mental health services in China. The results however need to be viewed with caution on account of the limited sample size.

### Structured cognitive interventions in a young population

Cognitive dysfunctions are central features of psychotic disorders. They often occur in the prodromal period of psychosis and continue through the course of illness (23, 24). The impairments significantly contribute to functional decline (25, 26). Cognitive training and remediation (CTR) interventions are useful to improve both cognitive and functional outcomes as suggested by several studies in chronic psychotic disorders (27). A meta-analysis of CTR in early psychosis, which included patients at high risk but had not yet developed a psychotic disorder, found small to moderate effects on cognition and functioning (28). However, in this analysis, intervention components varied considerably in the included studies and this may have affected the results. A more recent review of CTR literature in first-episode psychoses showed promising effects of a wide range of CTR interventions for cognitive impairments, although the functional improvement was less consistent (29). This volume presents two studies using CRT in a young population. Interest in the use of CRT reflects evidence of effectiveness and the need to test the implementation in real-life situations.

Chong et al. using a naturalistic design, discuss the application of cognitive remediation training (CRT) in patients with first-episode psychoses population in Singapore. Participants were part of the Singapore Early Intervention Psychoses program. They underwent 24 CRT sessions, using Cogpack and Neuropsychological Educational Approach to Remediation. Pre- and post-assessments were done using the Montreal Cognitive Assessment and Brief Assessment of Cognition in Schizophrenia. The participants significantly improved in several cognitive domains such as verbal memory, digit sequencing, and symbol coding. The study showed that CRT interventions were helpful in early psychosis.

Using a combined social cognitive remediation treatment with a neurocognitive remediation therapy, for young people with schizophrenia, bipolar disorder, or depression, the ADVANTAGE trial (Harris et al.), aimed to assess if functional outcomes improved. Neuropsychological Educational Approach to Remediation (NEAR) and the Social Cognition and Interaction Training (SCIT) treatments were delivered by trained therapists to participants randomized to either NEAR+ SCIT or NEAR + treatment as usual (TAU) groups. The trial was conducted over 20 weeks in five youth mental health services. Although the COVID pandemic restricted the follow-up, this research conducted in a naturalistic service setting, indicated that the group receiving a combined NEAR + SCIT approach showed a trend toward improved functioning.

### Interventions for families

Families of persons with mental disorders face a variety of challenges. Difficulties in coping with the problem, stigma, burden, and changes in role functions are some examples. Several types of interventions have been employed. Psychoeducation, stress reduction, emotional processing, cognitive reappraisal, and structured problem-solving are evidence-based family therapies.

Within the early intervention services, family interventions include psychoeducation, learning communication skills, and problem-solving. Multi-Family Therapy (MFT), for family caregivers of persons with mental illnesses, is an evidence-based family intervention that encourages mutual support and learning between families. MFT is provided as part of the Singapore EPIP services. The experiences of families that were part of Multi-Family Therapy (MFT) have been reported by Loh et al. A focused group discussion with families and clients who completed the MFT, was undertaken. The data from the discussion converged into themes such as processes that resulted in positive changes in family relationships and the progress in coping with psychosis. Families also suggested structural changes to the MFT. It was also noted that family members more than clients, preferred the therapist offered expert advice, this study proved that MFT could be adapted for intervention with Singaporean families. Changes in family relationships and coping strategies were the therapeutic processes that played a key role. The study suggests the need for therapists to adopt a flexible approach to working with families.

## Novel interventions

### Introducing a new intervention into mental health setting

Several reviews and reports have shown that over two-thirds of persons suffering from chronic psychoses do not have access to specialized mental health treatments (30, 31), especially in middle and lower-middle-income countries.

Based on the findings of a situational analysis, Bird et al. describe the prevailing psychosocial interventions in the existing services in two LMICs-India and Pakistan. The situational analysis revealed that a wide variety of psychosocial interventions were delivered to persons with psychosis in mental health facilities in a collaborative and integrated manner. These interventions were perceived positively by the respondents, who included patients, their family members, and mental health professionals. The data also revealed several real-world and systemic barriers to the services.

The investigation also explored the factors that were associated with introducing a new psychosocial intervention, DIALOG+. This app-based intervention, delivered *via* a tablet, computer, or smartphone is a psychosocial intervention, to enable the therapeutic effectiveness of usual meetings between patients and clinicians. Earlier RCTs, using DIALOG+, in both high and low-income countries, have demonstrated improvement in quality of life and reduction in symptoms (32, 33). In the current situational analysis, the use of a device to deliver new psychosocial interventions was noted to be both a barrier and a facilitator. There were some apprehensions about the use of technology. The authors emphasize the necessity to consider both the enthusiasm and the barriers when designing a new psychosocial intervention for the treatment of persons with psychosis within LMICs.

### Role of animals as therapeutic adjuncts

It is well known that the company of animals relieves and reduces the tension and stress of everyday life in human beings. More recently, research in animal-assisted interventions has been gaining ground. Using pets as an integral part of the treatment program, Animal Assisted Therapy (AAT) intends to improve a range of functioning in multiple domains (34). Studies testing an intervention that has employed animals have been heterogenous, methodologically inadequate, focused on the elderly, children, or psychiatric in-patients. Few RCTs, small sample sizes, and lack of control groups are some common limitations. Although AAT or AAA for in-patients seemed harmless and helpful for a range of diseases, questions about, the types of intervention, adverse events, cost, and disorders that would greatly benefit from these programs, remains unclear. A standardized set of outcome measures are needed to provide evidence for the potential use of such interventions (35).

Chen et al. aimed to test the potential of the use of Animal-assisted therapy (AAT). Using an RCT design, the study assessed the efficacy of AAT psychological intervention with dogs in a clinical setting. Patients with chronic schizophrenia who were middle-aged and older participated in a 12-week program. The outcome measures included the severity of symptoms and stress levels of the patients. Using a robust methodology, the researcher reported a reduction in psychiatric symptoms and stress. The study demonstrated the usefulness of AAT as an adjunct therapy to usual treatment programs.

### Co-production – A concept in need of a voice

Mental health service users do better when their choices and preferences are integrated into decisions for their management (36). An informed and respectful dialogue with service users is critical to decision-making about treatment options (37). Mental health service users were reported to approve a “shared” decision-making process, in a study investigating user preferences and understanding of the construction of decisions in community mental health. The concept of “shared” was seen as first prioritizing autonomy, and if that was not possible, deferring to advice from case managers. Service users opined that relationship and affective components of decision-making were more important than information-gathering or discussions on choices (36). The landmark Salzburg Statement on Shared Decision Making, stressed that both patients and clinicians be co-producers of health, encouraging patients to play a more dynamic role in the decisions regarding their healthcare needs as key stakeholders in healthcare (38). Co-production has been proposed to be the “third sector” to promote a shared process of decision-making (39). Commenting on co-production, a novel and burgeoning approach to mental healthcare service delivery, Lee et al. argue that co-production is a moral obligation. The authors urge that co-production needs to be an integral part of any recovery-oriented interventions in the mental health care system.

### Peer support – An under-used resource

A person with lived experience supporting others with mental illness is an established peer support intervention. The earliest investigation, UPSIDES-RCT a, parallel-group, multicentre, randomized controlled trial was designed to test the effectiveness of using peer support to develop empowering mental health services (UPSIDES) at several levels (40).

Will such intervention be feasible in low and middle-income countries? A cross-sectional survey in an outpatient mental health clinic in Chennai, South India (Sims et al.), aimed to understand if interventions by peer volunteers were acceptable and feasible. Persons diagnosed with schizophrenia, their family caregivers, and mental health professionals responded to questions on the usefulness of peer support interventions and the various areas that peers could provide the services. The majority of the participants welcomed peer support interventions. The areas of interventions suggested included practical help for persons with schizophrenia, to improve their mental health and their daily routines. There was an emphasis on independent living, social relationships, and meeting medication and treatment-related goals. Highlighting the potential acceptability of peer volunteer programs, the authors emphasized the need to test the effectiveness of implementing a peer support program as a necessary component of a mental health service.

### Interventions using smartphones – Potential for volunteering

Research on groups with positive attitudes and behaviors toward people with mental illness who volunteer in mental health services is sparse (41). A systematic review with data on 540 volunteers suggested that volunteers were a heterogeneous group, largely comprised of females. Volunteers reported feeling good about their involvement in mental health care. Persons with a psychiatric illness benefitted from a “friend”, who was non-discriminating and hands-on in augmenting social involvement (42). Several other meta-analyses have reported similar findings (43). However, existing volunteering programs have been noted to lack flexibility and do not regard people's preferences and challenges. This emphasizes a need to integrate technology into everyday life.

The “Phone Pal” intervention was designed to investigate whether volunteering telephonically would work for people with psychosis, using several approaches like phone calls, videoconferencing, messaging, or e-mails). da Costa et al. report on a protocol to test the feasibility of the intervention, using a mixed methods approach, in the United Kingdom. The protocol explored the feasibility of recruitment, retention of participants, methods of data collection, and modes of communication. Outcome measures from patients and volunteers at baseline and follow-up, included quality of life, level of physical activity, change in self-esteem, and social comparison. A ranking of perception of their relationship with each other was also gathered at follow-up. The researchers hoped to establish the acceptability of this intervention and to evaluate the feasibility of conducting a trial.

## The role of mental health setting when delivering new interventions

Two studies reported in this Research Topic, bring to light the role of the mental health setting as critical consideration when delivering a new intervention. Any new psychosocial intervention needs to consider both the enthusiasm of the professionals as well as barriers in the designing phase. The situational analysis (Bird et al.) within LMICs reflected on the existing practices in the two settings. It explored contact with services, access to new cases, follow-up patterns, the multidisciplinary approach, and the contribution of users and family members to the program. The emphasis of the situational analysis focused on an exploration of the barriers and facilitators, including organizational readiness for a new intervention.

Fibbins et al. demonstrated the feasibility of implementation of an exercise physiology clinic for users within the service. They opined that such services would increase the levels of physical activity and reduce inactive times for persons with chronic mental illness. They also reiterated the need for a collaborative approach in implementing an exercise clinic and taking into consideration the individual needs of the patients.

## Conclusion

This Research Topic has highlighted the wide range of interventions that have been an intervention. Consistent with a recent systematic review, combined with a theory-driven logic model approach, the interventions described in this collection of articles, can be seen to align with four fundamental requirements that include the provision of information and skills, promotion of a working alliance, role modeling recovery and increase in choices (3). While many of the interventions replicated existing interventions, adapting these to their settings, several novel strategies have been reported. All these interventions have been designed using sound methodologies, adapting to the culture of the population under study and the setting in addition to lending themselves to replication. It is noteworthy that researchers have considered populations across the illness span and chronicity of psychotic illnesses. Establishing the role of peer volunteers in the LMIC setting has a strong implication for adding to the workforce of mental health professionals. There is a paucity of biological correlates that may explain the effectiveness of the interventions, a need that needs to be addressed in future studies.

## Author contributions

All authors listed have made a substantial, direct, and intellectual contribution to the work and approved it for publication.
